# A Very Rare Combination: two Scimitar Veins and a Myocardial Bridge

**DOI:** 10.21470/1678-9741-2018-0240

**Published:** 2020

**Authors:** Freddy Ponce Tirado, Nora Pierina Fernandez Faconi, Ricardo Barros Corso, Isaac Azevedo Silva

**Affiliations:** 1 Clinica Delgado Auna - Cardiac Surgery, Lima, Peru.; 2 Cardiovascular Associados, Brasília, Distrito Federal, Brazil.

**Keywords:** Scimitar Syndrome. Vena Cava, Inferior. Coronary Vessels, Lung, Vascular Malformations, Drainage

## Abstract

Scimitar syndrome is a rare congenital anomaly characterized by partial or complete anomalous pulmonary venous drainage of the right (rarely left) lung into the inferior vena cava. This anomalous vein resembles the curved Turkish sword “scimitar”^[1]^. Only few cases were reported with two scimitar veins^[2]^. “Myocardial bridge” constitutes a portion of the myocardial tissue that bridges a segment of the coronary artery, mostly the left anterior descending coronary artery . For the first time, a combination of double scimitar vein and a myocardial bridge was described in this study.

**Table:** 

Abbreviations, acronyms & symbols	
APC	= Aortopulmonary collaterals
CT	= Computed tomography
EKG	= Electrocardiogram
IVC	= Inferior vena cava
LADCA	= Left anterior descending coronary artery
MB	= Myocardial bridge
RPA	= Right pulmonary artery

## INTRODUCTION

Scimitar syndrome is a rare congenital heart disease first reported by Cooper^[3]^ in early 1800 and named by Neill in 1960^[4]^. It consists of a rare association of congenital heart and lung abnormalities, features anomalous pulmonary venous return to the inferior vena cava, typically from the right lung^[5]^. The scimitar vein most often descends anterior to the hilum, grows larger as pulmonary veins drain into it, and curves slightly to the left to join the inferior vena cava (IVC). This creates a distinctive shape that resembles a curved Turkish sword, or scimitar. It occurs more commonly in females and is occasionally familial. The left lung is very rarely involved and the reason for this is unknown. The true incidence of this condition is unknown since the syndrome may remain undetected in asymptomatic patients who do not undertake images. Scimitar syndrome overlaps with pulmonary sequestration and the term venolobar syndrome has been coined to include these associated pulmonary and vascular malformations^[6,7]^. Associated findings classically include atrial septal defects, aortopulmonary collaterals (APC), and hypoplasia of both the right pulmonary artery (RPA) and right lung^[5]^.

“Myocardial bridge” (MB) constitutes a portion of the myocardial tissue that bridges a segment of the coronary artery, mostly the left anterior descending coronary artery (LADCA). MB is also known as muscular bridge, intramural coronary artery, mural coronary, tunneled coronary artery, or myocardial loop. MB was firstly described in 1737 by Reyman^[8]^. The detection of the MB may vary according to the method: by angiography is 0.4%-15.8%, by computed tomography 3.5%-58%, and by autopsy 4.7%-60.0%^[9]^.

Although many congenital heart defects were associated with scimitar, none reported case was found with double scimitar vein and myocardial bridge.

## CASE REPORT

**Fig. 1:**
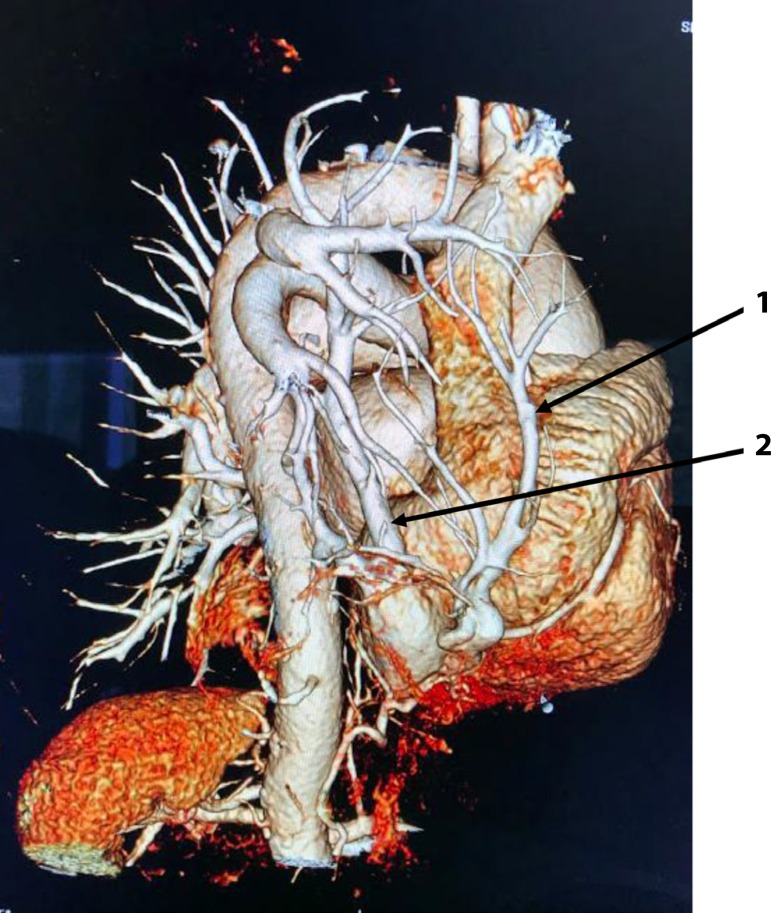
1,2=right scimitar veins.

**Fig. 2:**
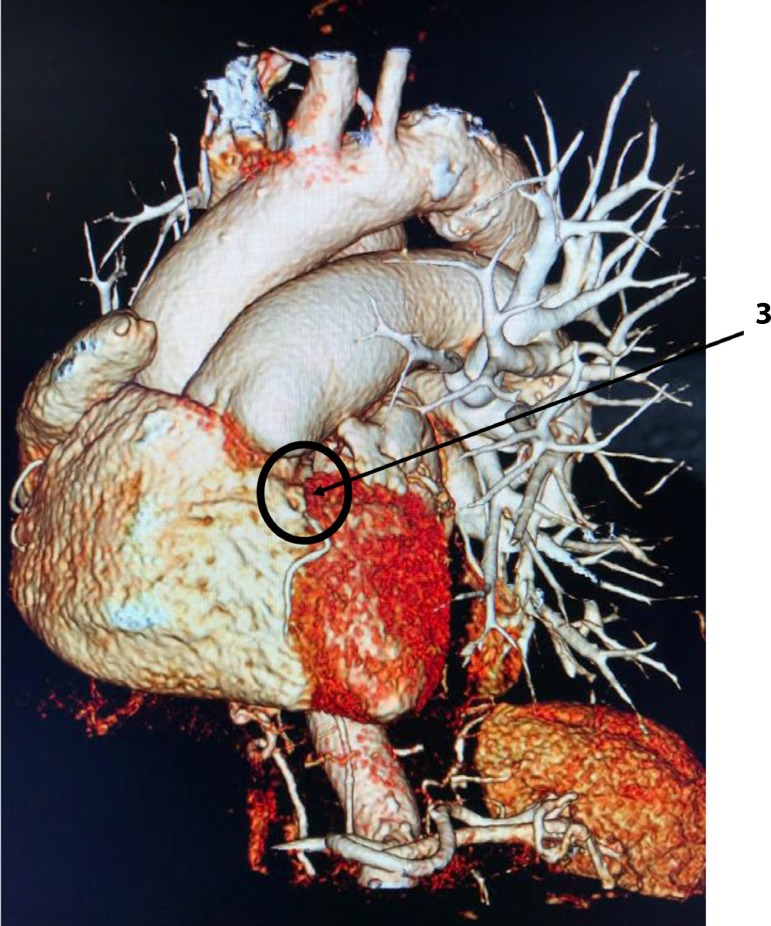
3=proximal LAD myocardial bridge.

**Fig. 3:**
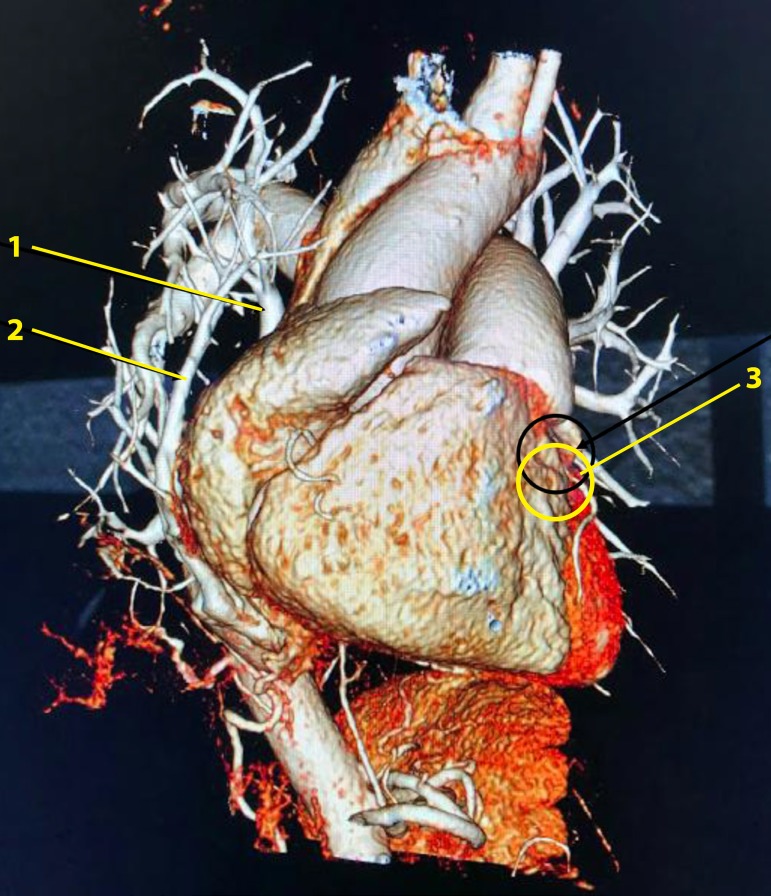
1,2=right scimitar veins; 3=LAD myocardial bridge.

A 76-year-old white female patient has asked for medical attention in an emergency department, referring a retrosternal discomfort with a previous history of self-limited tachycardia, chest pain and orthopnea. The patient was very anxious when arrived and informed a history of asthma. Her non-invasive measured arterial blood pressure was 160x110mmHg and 90 bpm was her cardiac frequency, at that moment. The electrocardiogram (EKG) showed a normal sinus rhythm, although she had a previous exam showing an atrial fibrillation with 150 bpm. At clinical chest examination, a reduced murmur was found in the right hemithorax and an increased intensity of P2. The initial clinical suspicion was pulmonary artery embolism. A chest x-ray was performed and an image of a vascular structure coming from the top of the right lung till the diaphragm was identified, suggesting an anomalous pulmonary vein drainage (Scimitar Syndrome). To confirm this clinical and radiological hypothesis chest and cardiac contrast-enhanced computed tomography (CT) were ordered. The thorax CT revealed partial anomalous pulmonary venous return with upper and lower right pulmonary veins draining into IVC (two Scimitar veins) and right lung hypoplasia (Figure 1). Concomitant contrasted cardiac tomography showed an intramuscular course of the proximal descending artery (myocardial bridging) (Figure 2,3). She was treated clinically with complete remission of her symptoms. Considering the advanced age of this woman and her good response to clinical management, we did not proceed to surgical corrective approach. She is now in optimized clinical treatment with calcium channel blocker, bronchodilators and oral anticoagulation (history of atrial fibrillation) without decompensation either re-hospitalization.

## DISCUSSION

Patients with scimitar syndrome may be seen in infancy, childhood or adulthood. The clinical spectrum ranges from severely ill infants to asymptomatic adults, since, infants typically have features of congestive heart failure from a significant left to right shunt. In adults, the diagnosis may be difficult, because of the wide range of symptoms at clinical onset. After diagnose confirmation in the adulthood, owing to the rarity of scimitar syndrome, there are insufficient data to draw conclusions regarding indications for medical versus surgical management and, obviously, there are no guidelines for surgical correction. Usually, the magnitude of symptoms, patient desire and response to conservative management will dictate the approach^[5,9,10]^.

MB may provoke symptoms of coronary heart disease by direct compression of the LADCA by the myocardial contraction and by induction of coronary atherosclerosis in the LADCA segment proximal to the MB^[9]^. Thereby, angina, myocardial ischemia, myocardial infarction, and even sudden death are considered as bridging complications. For symptomatic patients, three options of treatment were described: medication, stent placement in the bridged segment, and surgical treatment. Although the beta-blockers are the first line to pharmacological treatment, in this particular case, e calcium channel blockers were used due to previous history of asthma. As for scimitar syndrome, no guidelines are available, and, the anatomical properties of the MB will influence the treatment choice.

Our patient is unique due to the presence of two scimitar veins and a LADCA myocardial bridging and became asymptomatic with optimized medical treatment.

## CONCLUSION

To our knowledge, this is the first time that both, double scimitar vein and myocardial bridging were described together. The rarity of the double scimitar vein by itself is worthy to communicate. When we noticed this scarce condition in an unusual association, more motivated we became. Considering the time spent to diagnose an important pulmonary malformation in this patient (up to seven decades!) this report may sound as an alert to our colleagues.

**Table:** 

Authors' roles & responsibilities	
FPT	Substantial contributions to the conception or design of the work; or the acquisition, analysis, or interpretation of data for the work; final approval of the version to be published
NPFF	Substantial contributions to the conception or design of the work; or the acquisition, analysis, or interpretation of data for the work; final approval of the version to be published
RBC	Substantial contributions to the conception or design of the work; or the acquisition, analysis, or interpretation of data for the work; final approval of the version to be published
IAS	Substantial contributions to the conception or design of the work; or the acquisition, analysis, or interpretation of data for the work; final approval of the version to be published
